# Ketamine Administration Leads to Learning-Memory Dysfunction and Decreases Serum Brain-Derived Neurotrophic Factor in Rats

**DOI:** 10.3389/fpsyt.2020.576135

**Published:** 2020-09-25

**Authors:** Miao Li, Aiming Xie, Ya Liu, Qian Zeng, Shucai Huang, Qiuping Huang, Tianli Shao, Xinxin Chen, Zhenjiang Liao, Yi Cai, Zhijie Xiao, Xiaojie Zhang, Hongxian Shen

**Affiliations:** ^1^ Department of Psychiatry, The Second Xiangya Hospital of Central South University, and National Clinical Research Center for Mental Disorders, Changsha, China; ^2^ Institute of Mental Health of Central South University, Chinese National Technology Institute on Mental Disorders, Hunan Key Laboratory of Psychiatry and Mental Health, Hunan Medical Center for Mental Health, Changsha, China; ^3^ Department of Neurology, The Second Xiangya Hospital, Central South University, Changsha, China; ^4^ Department of Neurology, The First Hospital of Changsha, Changsha, China; ^5^ Affiliated Wuhan Mental Health Center, Tongji Medical College of Huazhong University of Science & Technology, Wuhan, China; ^6^ Department of Neurology, Changsha Central Hospital, Changsha, China; ^7^ Department of Psychiatry, The Fourth People’s Hospital of Wuhu, Wuhu, China; ^8^ Department of Psychiatry, Geriatric and Somatic Diseases, Brain Hospital of Human Province, Hunan University of Chinese Medicine Clinical College, Hunan Mental Behavior Disorder Research Center, Changsha, China

**Keywords:** ketamine administration, spatial learning, memory, Morris water maze, brain-derived neurotrophic factor

## Abstract

**Objective:**

This study investigated the effects of acute or chronic ketamine administration on learning and memory function as well as levels of brain-derived neurotrophic factor (BDNF) in the hippocampus and blood in order to explore the potential correlation between learning-memory dysfunction and ketamine.

**Methods:**

Rats were treated with 25 mg/kg ketamine for 3 d (n = 20) or 14 d (n = 20). Saline-treated rats were used as controls. The Morris water maze test was used to evaluate spatial learning and memory after 10 d of withdrawal. The level of BDNF in serum and the hippocampus were measured by ELISA.

**Results:**

The number of platform crossings and residence time in the target platform quadrant were significantly reduced in ketamine 3 d and 14 d groups than in the saline controls (both *p* < 0.05). In addition, the average escape latency of ketamine 3 d and 14 d groups were significantly longer than that of the saline 3 d and 14 d groups (*p* < 0.0001), respectively. Further examination found that only serum samples from ketamine 14 d group showed significantly decreased BDNF level compared to that from saline 14 d groups (*p* < 0.05). However, no differences were detected in hippocampus samples.

**Conclusion:**

Chronic ketamine exposure (25 mg/kg) causes spatial learning and memory deficits in SD rats, which may be associated with decreased serum BDNF levels.

## Introduction

Ketamine is a commonly studied psychoactive substance and known drug of abuse. Recently, it has been widely abused as a new type of drug (also known as K powder) due to its addictive properties (1, 2). Ketamine abuse can lead to cognitive impairment (3, 4). Ketamine is an antagonist of the N-methyl-D-aspartate (NMDA) receptor, and can lead to similar symptoms of addiction (5). Recent studies have shown that low-dose ketamine does not have a significant effect on cognition function (6), but sub-anesthetic dose and anesthetic-dose ketamine could cause cognitive impairment by affecting executive function, procedural function, episodic memory, semantic memory, and working memory (2, 6, 7). Importantly, the effects of ketamine vary greatly with duration. In humans, acute ketamine treatment is known to impair episodic and working memory, recognition memory, and procedural learning (7). This has been further confirmed in mice, as chronic ketamine treatment also leads to memory impairment (8). Ketamine can induce behavioral changes in users and this change is related to the frequency and dose of use (9).

Brain-derived neurotrophic factor (BDNF), abundant in the central nervous system, is known to regulate synaptic plasticity as well as participate in learning and memory processes. BDNF is also likely to participate in the pathophysiology of drug addiction, including the cognitive impairment (10–12). In a study by Ke et al. serum BDNF concentrations were significantly lower in chronic ketamine users than in health controls (2). Importantly, it has been found that the level of BDNF in blood can be used to approximate protein concentration changes in brain tissues (12). It is therefore likely that BDNF concentrations are similarly lower in the brain of chronic ketamine users, particularly within the reward system of the brain. This network of brain structures, namely the ventral tegmental area of the midbrain and its connections to the nucleus accumbens and prefrontal cortex, governs reward, and is well established to be involved in drug addiction (13). In addition to reward circuity, exposure to drugs of abuse, including ketamine, can alter plasticity in cognition as well as learning and memory-related brain areas. Importantly, these changes can persist long after drug exposure has ceased (14).

Therefore, in order to further elucidate the potential relationship between BDNF and ketamine-induced learning-memory dysfunction, we investigated the effects of acute and chronic ketamine (25 mg/kg) on cognition as well as BDNF levels in the blood and hippocampus of Sprague-Dawley (SD) rats.

## Materials and Methods

### Animals

A total of 75 adult healthy male SD rats were randomly divided into four groups: ketamine 3 d group (n = 20), saline 3 d group (n = 18), ketamine 14 d group (n = 20), saline 14 d group (n = 17). The experiment was carried out after a week of adaptive feeding.

### Ketamine Exposure

All rats were intraperitoneally injected with ketamine, 25mg/kg for either 3 d or 14 d, and the saline group was injected with 0.9% saline for 3 d or 14 d as controls. Injections were performed daily at 4:30 p.m. In order to avoid the effect of withdrawal reaction on behavioral experiments, the rats were given a 10-day withdrawal period.

### Morris Water Maze (MWM) Test

MWM test was conducted in a round stainless steel water tank with a diameter of 1.2 m and a height of 0.5 m, with an attached tracking and recording camera using SMART V2.5.21 program software (Panlab, Spain). A submerged transparent platform was placed 30 cm from the pool’s edge and submerged 2 cm beneath the water surface. The water temperature was controlled at 22–24°C. The platform remained in the same position throughout trials.

The platform was removed, and the rats were free to swim for 2 min to screen out the insensitive rats before the start of the experiment. As a result, only 75 healthy SD rats were selected for further experiments. The selected healthy rats were tested for the navigation experiment and the space exploration experiment.

Positioning navigation experiment: Rats were trained for 4 consecutive days to determine their learning ability. At the beginning of the training, starting points were randomly selected in the southeast and the northwest quadrants, and the rats were placed gently against the pool wall. A video system tracked and recorded the rats swimming in the maze. The time taken to find the platform was recorded as escape latency. If rats failed to reach the platform within 60 s, the escape latency was recorded as 60 s, and the rats were guided to the platform and left for 10 s. Each rat was trained 4 times a day, 20-30 min for each interval, and the average latency across these trials was used as the latency value for each day. On the 5^th^ day, each animal was tested in four, single 60 s sessions from each quadrant.

Space exploration experiment: the main purpose was to measure the spatial memory ability of rats. After the 5th day of the water maze experiment, the platform was removed allowing the rats to swim freely for 60 s. The duration spent in the target quadrant and the number of crossing times in the quadrant were recorded

### Material and Tissue Processing

Blood samples (4 ml) were collected from the inferior vena cava after abdominal anesthesia with 10% chloral hydrate (0.3 ml/100 g). All serum samples were separated, and stored in a -70°C ultra low temperature freezer for later use.

All rats were then sacrificed to harvest hippocampus. Pre-chilled physiological saline was added to obtain 10% hippocampal homogenate, and proteins extracted using RIPA lysates. 1 ml RIPA lysate (containing 1mmol PMSF) was mixed with 100 μg of sample and sonicated for 1 min (sonicated 2 s and interval 1 s) in a 1.5 ml EP tube on the ice. All samples were then centrifuged at 2,500 r/min for 10 min at 4°C to harvest supernatant, and stored in a -70°C ultra low temperature freezer before use.

### Detection of BDNF Concentration by ELISA Method

Levels of BDNF were determined using Thermo Scientific’s Rat BDNF ELISA kit per the manufacturer’s instructions. Briefly, the sample (100 µl) was added to its appropriate well, and incubated for 2.5 h at room temperature. The solution was discarded, and the wells were washed 4 times with 1× Wash Buffer. Next, 100 µl of 1× prepared biotinylated antibody was added to each well and incubated for 1 h at room temperature with gentle shaking. 100 µl of prepared Streptavidin-HRP solution was then added to each well and incubated for 45 min at room temperature with gentle shaking. The solution was than discarded and the wells were washed 4 times with 1× Wash Buffer. Finally, 100 µl of TMB Substrate was added to each well and incubated for 30 min at room temperature in the dark with gentle shaking. Each plate was evaluated within 30 min of stopping the reaction. Optical density OD values were measured at the wavelength of 450 nm, and the OD values of supernatant and serum in hippocampal homogenate were measured using a multi-function enzyme labelling analyzer.

### Ethics Statement

All experiments were conducted in accordance with the animal experiment regulations of the Second Xiangya Hospital Ethics Committee of Central South University. The protocol was approved by the Second Xiangya Hospital Ethics Committee of Central South University.

### Statistical Analysis

The statistical methods were analyzed by SPSS 22.0 software. One way ANOVA was used to analyze the data in accordance with normal distribution and homogeneity of population variance. Two way ANOVA and Bonferroni’s multiple comparisons test were used to analyze the data of escape latency in water maze. LSD method was used for comparison between groups. The measurement data were expressed as mean ± standard deviation (X ± SD) or means ± sem. For data not in accordance with normal distribution, Mann Whitney U method in rank sum test was used for inter group comparison, and the results were expressed by relevant parameters. P represents the significant level of the test, P < 0.05 means the difference is statistically significant.

## Results

### Quantitative Analysis of Experimental Animals

All 75 rats (ketamine 3 d group n = 20, saline 3 d group n = 18, ketamine 14 d group n = 20, saline 14 d group n = 17) were included in the statistical analysis of the experimental results. There were no accidental deaths in the experiment. A total of 75 rats were screened by water maze.

### Experimental Results of Morris Water Maze

**Figure 1:**
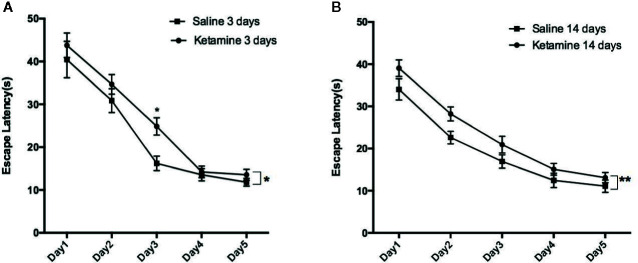
The escape latencies of rats in water maze positioning navigation experiment. Data shown as means ± sem. Rats were grouped by different treatment, Two-way ANOVA with Bonferroni correction were used to determine whether difference existed based on experience level. Significantly difference were seen between saline 3 days group vs. ketamine 3 days group as shown in **(A)**, saline 14 days vs. ketamine 14 days group as shown in **(B)**. Further analysis each group at different time point by Bonferroni’s multiple comparisons test in Prism6, the significant difference showed in day3 comparing saline 3 days group with ketamine 3 days group. *p<0.05, **p<0.0001.

The results from the Morris water maze are shown in **Figure 1** and **Table 1**. The average escape latency of ketamine 3 d and 14 d groups were significantly longer than that of the saline 3 d and 14 d groups (*p* < 0.0001), respectively. The significant difference showed in day3 comparing saline 3 days group with ketamine 3 days group by multiple comparison (p<0.05).

**Table 1:** Statistical results of the number of crossing platforms and residence time in each group of rats in space exploration experiments (X ± SD).

Group	Number	The number of crossing platform	The residence time in the target platform quadrant(s)
ketamine 3 d	20	6.5 ± 2.0^bd^	6.7 ± 3.5^bd^
saline 3 d	18	8.3 ± 3.5^a^	9.6 ± 4.0^a^
ketamine14 d	20	8.0 ± 2.8^d^	8.5 ± 3.7^d^
saline 14 d	17	10.0 ± 2.7^ac^	11.0 ± 3.7^ac^

^a^P value comparison with the ketamine 3 d group, P < 0.05, ^b^P value comparison with the saline 3 d group, P < 0.05, ^c^P value comparison with the ketamine 14 d group, P < 0.05, ^d^P value comparison with the saline 14 d group.

On day six, in the space exploration experiment, a significant reduction in the number of platform crossings as well as residence time was observed in the ketamine 3 d group compared to the saline 3 d group (*p* < 0.05). Compared to the saline 14 d group, the number of platform crossings and the residence time was also significantly reduced in the ketamine 14 d group (*p* < 0.05). However, there was no significant differences observed between the ketamine 3 d and ketamine 14 d groups.

**Figure 2:**
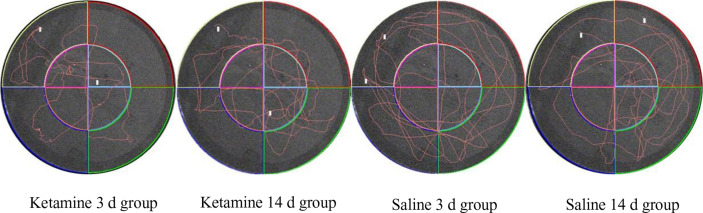
Typical trajectory map of four groups of rats in space exploration.

As showed in **Figure 2**, the track map of the target platform quadrant in the 3 d ketamine group is thinner than in the 3 d saline group. Similar results were also observed between the ketamine 14 d group and the saline group. Therefore, as observed in both the 3 d and 14 d group, there appears to be a memory deficit in the ketamine group when compared to saline controls.

### Results of BDNF Concentration in Serum and the Hippocampus

The serum concentration of BDNF in the ketamine 14 d group was significantly lower than that of the saline 14 d group (*p <*0.05). No significant difference was observed in serum BDNF concentration between the ketamine 3 d and saline 3 d group (**Table 2**).

**Table 2:** Comparison of serum brain-derived neurotrophic factor (BDNF) concentration of each group.

Group	Total number	U	W	Z	P
A vs B	38	171.000	342.000	-0.263	0.792
C vs D	37	88.000	299.000	-2.469	0.014^*^
A vs C	40	183.000	393.000	-0.460	0.646

Group A represents ketamine 3 d group, Group B represents saline 3 d group, Group C represents ketamine14 d group, Group D represents saline 14 d group, *indicates ketamine 14 d group compared with saline14 d group, P < 0.05. U, W, and Z value represent statistics. P represents significant difference.

BDNF concentrations in the hippocampus are showed in **Table 3**. No significant differences were found in hippocampal BDNF concentrations between any of the ketamine and saline groups (ketamine 3 d vs. saline 3 d: ketamine 14 d vs. saline 14 d).

**Table 3:** Comparison of hippocampus brain-derived neurotrophic factor (BDNF) concentration of each group.

Group	Total Number	U	W	Z	P
A vs B	38	175.000	385.000	-0.146	0.884
C vs D	37	124.000	334.000	-1.402	0.161
A vs C	40	199.000	409.000	-0.027	0.987

Group A represents ketamine 3 d group, Group B represents saline 3 d group, Group C represents ketamine14 d group, Group D represents saline 14 d group. U, W, and Z value represent statistics. P represents significant difference.

## Discussion

This experiment was designed to explore the neural mechanism related to ketamine addiction. Studies have found that ketamine abuse can lead to increased apoptosis of neurons in the frontal lobe, temporal lobe, hippocampus, among other regions, affecting cognitive function (1). Given that ketamine addiction can produce behaviors similar to schizophrenia, it has been suggested that ketamine addiction can be used as an experimental model of schizophrenia. Therefore, we assessed for differences in hippocampal and serum BDNF levels simultaneously to further elucidate the potential relationship between BDNF and ketamine-induced learning-memory dysfunction.

We first determined the effect of the acute and chronic ketamine (25mg/kg) exposure on learning ability and memory in rats. Our results showed that the continuous use of ketamine for 3 d or 14 d both lead to learning and memory dysfunction. The concentration of BDNF in serum and hippocampal homogenate was also measured by ELISA. Interestingly, we only observed a significant difference in serum BDNF concentrations between the ketamine 14 d group and the saline 14 d group. Based on this, we further assessed the effect of ketamine on learning-memory dysfunction in rats and its relationship with BDNF.

A recent study showed that the effect of ketamine on cognition was directly related to the dosage (6, 15, 16). However, there is no specific unity in the classification of low dose, subanesthetic dose and anesthetic dose among the present experimental literature. Low dose (2.5–10 mg/kg) ketamine can cause cognitive impairment, while lower doses (< 2.5 mg/kg) and a slightly higher dose (10–20 mg/kg) do not significantly affect cognition after 10 consecutive days of exposure (16). Some researchers have even used higher doses of ketamine (25 mg/kg) to study the effects on rats and found that they lead to positive and negative symptoms similar to schizophrenia as well as affect cognitive function (17). However, Imre et al. (9), suggest that 4–16 mg/kg of ketamine is a subanesthetic dose for rodents, and 20–50 mg/kg is a high dose (18). Meanwhile, Newcomer et al. have shown that for rats, the minimum response threshold is 40 mg/kg (19). Previous studies have investigated the effect of ketamine on memory consolidation, and found that subanesthetic doses (5–50 mg/kg) did not affect the reconsolidation phase, whereas higher, anesthetic doses (125–150 mg/kg) did affect memory reconsolidation (15). Given the above findings, we chose the 25 mg/kg dose to study the effects of ketamine on learning and memory in rats. Consistent with previous studies, our results show that in the water maze experiment, the average latency of the ketamine-14 d group was longer than that of the saline-14 d group. The number of platform crossings and the residence time of the rats in the 3 d and the 14 d ketamine groups were lower than that of the control groups. These results suggest that repeated exposure to 25 mg/kg ketamine negatively affects learning and memory in SD rats.

It has also been reported that the effect of ketamine on cognition is related to the duration of exposure as well as the time since last exposure. Single ketamine exposure and repeated ketamine exposure both cause cognitive impairments (10, 20). For example, when ketamine (30 mg/kg) was injected continuously for one month, the spatial memory ability of rats was significantly reduced (21).

In animal studies, a single exposure as well as 3–5 d of exposure have all been defined as acute ketamine exposure (8, 10, 16, 22). Likewise, the definition of chronic ketamine exposure varies between experimental studies. In fact, some have defined chronic ketamine exposure in rodents as 5 d (2), 7 d (17), 10 d (16), 14 d (23), and 30 d (21). Therefore, based on the literature, we defined 3 d ketamine exposure as “acute” and 14 d as a “chronic” in order to study the difference in cognition between acute and chronic ketamine exposure in rats.

The half-life of ketamine is about 1 to 2 h, and the withdrawal reaction usually takes place between 0.5 d to 3 d (2). Most previous studies conducted the behavioral test 24 h after the last drug injection, thus not accounting for the effects of the withdrawal response. In order to avoid the effect of acute withdrawal in this experiment, the rats were given ketamine for 10 d after the last exposure. This study showed that acute and chronic ketamine (25 mg/kg) exposure both lead to learning and memory dysfunction in rats.

It had been reported that no matter acute or chronic exposure to ketamine, the damage to learning and memory of rats is persistent and irreversible (16, 23). In this experiment, the learning and memory abilities of rats were measured 10 days after ketamine withdrawal. Both the acute or chronic ketamine exposure led to significant impairment in cognitive dysfunction, comparing to control group.

n human and primates in general, cognition is mainly controlled by the prefrontal cortex and hippocampus, while rodents mainly depend on the function of the hippocampus (24). Importantly, deficiencies in BDNF is associated with hippocampal neuronal apoptosis. To verify whether learning-memory dysfunction in rats caused by ketamine is associated with BDNF deficiency in the hippocampus, we determined the levels of BDNF in the hippocampus of ketamine-treated rats. Interestingly, we found that there was no significant change in hippocampal BDNF concentrations after ketamine withdrawal. Intriguingly, we found that levels of BDNF were reduced in serum after chronic ketamine withdrawal. This suggests that serum BDNF levels are a better indicator of learning-memory dysfunction produced by ketamine exposure than hippocampal BDNF levels.

In conclusion, our study demonstrated that chronic ketamine (25 mg/kg) administration causes spatial learning and memory impairment in SD rats, and that this deficit might be associated with decreased serum BDNF levels. Additionally, our results also suggest that there are other mechanisms regulating BDNF levels in the brain. Nevertheless, it is still unclear how deficiencies in serum BDNF levels lead to learning and memory deficits in rats, requiring further exploration.

The limitation of this study is that we did not study the effects of ketamine addiction on human cognitive function and serum BDNF concentration. In the future, cognitive impairment caused by different doses and time of ketamine addiction patients can be measured with cognitive scale, and serum BDNF concentration can be measured at the same time. In order to understand the changes of brain structure, functional imaging of brain can be completed at the same time to understand the changes of neurotransmitters in the brain.

## Data Availability Statement

The raw data supporting the conclusions of this article will be made available by the authors, without undue reservation.

## Ethics Statement

The animal study was reviewed and approved by the Second Xiangya Hospital Ethics Committee of Central South University.

## Author Contributions

HS, XZ, and ZX conceptualized the experiments. ML, YL, and QZ performed the experiments. XZ, SH, and QH undertook the statistical analysis. ML and AX wrote the first draft of the manuscript. XZ and YC reviewed and edited the manuscript. XZ, TS, XC, and ZL contributed to the final manuscript. All authors contributed to the article and approved the submitted version.

## Funding

This work was supported by the National Natural Science Foundation of China (81971249), the National Basic Research Program of China (2015CB553504), the National Research Program of China (2016YFC0800908-Z02), and the National Natural Science Foundation of China (81501108).

## Conflict of Interest

The authors declare that the research was conducted in the absence of any commercial or financial relationships that could be construed as a potential conflict of interest.
